# Integrating Insights: A Mixed‐Methods Approach to Nurses' Competencies in Evidence‐Based Practice and Clinical Decision‐Making

**DOI:** 10.1002/nop2.70525

**Published:** 2026-04-10

**Authors:** Seda Sarıköse, Fatma Sevim, Şebnem Alık, Pelin Karaçay, Özgen Yaşar

**Affiliations:** ^1^ Department of Nursing Koç University School of Nursing Istanbul Türkiye; ^2^ Koç University Semahat Arsel Nursing Education Practice and Research Center Istanbul Türkiye

**Keywords:** clinical decision‐making, evidence‐based practice, mixed methods study, nurse competencies

## Abstract

**Aim:**

To identify the factors influencing nurses' Evidence‐based practices (EBP) competencies and clinical decision‐making (CDM) levels.

**Design:**

A convergent parallel mixed‐methods study.

**Methods:**

The quantitative data were collected from 387 nurses via the EBP Competence Questionnaire and the Clinical Decision‐Making in Nursing Scale, while qualitative data were gathered from semi‐structured interviews with 20 nurses. Quantitative data were analysed using descriptive statistics, ANOVA, and multiple linear regression, and qualitative data were subjected to thematic content analysis.

**Results:**

Regression analysis revealed that higher education, research involvement, and following scientific publications positively influenced EBP competencies. Working in inpatient units and clinical nursing roles negatively affected EBP and CDM abilities. A significant positive correlation was found between EBP competence and CDM skills. Qualitative findings identified four themes: Implementation Areas of EBP, Competence in EBP, Impacts of EBP, and Facilitators and Barriers to EBP.

**Conclusion:**

Strengthening nurses' EBP competencies and CDM requires targeted strategies such as education, access to organizational resources and supportive policies.

**Implications for the Profession:**

Addressing barriers and fostering a culture of continuous learning can enhance patient care and nursing outcomes.

**Impact:**

By addressing gaps in EBP implementation and decision‐making skills, the findings serve as a benchmark for policymakers, educators and healthcare administrators to create supportive infrastructures, promote continuous professional development, and foster a culture of evidence‐based practice worldwide.

**Reporting Method:**

The study was reported in accordance with the GRAMMS guidelines.

**Patient or Public Contribution:**

No patient or public contribution.

## 1 Introduction

Evidence‐based practice (EBP) is an approach that combines the results of scientific studies with the expertise of researchers. This approach is fundamental to supporting scientific and critical decision‐making among nurses in the clinical decision‐making (CDM) process (Schaefer and Welton 2018). Decision‐making based on scientific knowledge is considered a defining feature of nursing professionalism (Watkins 2020), and the International Council of Nurses (ICN) has declared EBPs as a central professional responsibility of nurses (Hutton et al. 2016).

EBPs support personalized and holistic nursing care that addresses the specific needs of patients (Sapri et al. 2022; den Hertog and Niessen 2021) and improves the quality of care (Alatawi et al. 2020). Study results showed that, despite the recognized benefits, nurses face multiple barriers that hinder the effective implementation of EBP in clinical settings (Clarke et al. 2021; Golge et al. 2024; Naghibi et al. 2021). Identifying these barriers can enable healthcare systems to develop strategies and solutions that support the effective implementation of EBP (Alatawi et al. 2020). Also, nurses' EBP competencies, defined as the knowledge, skills and attitudes required to effectively integrate evidence into practice, and their CDM skills, referring to the ability to assess options, anticipate outcomes, and select appropriate interventions, are crucial to increasing evidence‐based uptake (Melnyk and Fineout‐Overholt 2022; Standing 2020). Therefore, this study aimed to examine nurses' EBP competencies and CDM levels and the factors affecting them.

## 2 Background

Despite the growing emphasis on evidence‐based care globally, recent reports indicate that the implementation of EBP among nurses remains limited. The World Health Organization (WHO 2017) highlights that nurses often face persistent barriers, including heavy workload, insufficient staffing, lack of organizational support, and limited access to resources, which hinder their ability to apply evidence in daily practice. Furthermore, the report underlines that many nurses have not yet acquired the necessary knowledge and skills to critically appraise and utilize evidence, resulting in a gap between positive attitudes towards EBP and its actual implementation in clinical settings.

Researchers have recently intensified their studies to understand why nurses base their decisions on sources other than scientific and research knowledge during clinical practice (Alatawi et al. 2020; Golge et al. 2024). According to a study, most nurses, including advanced practice nurses, could not perceive themselves as competent in implementing EBP in clinical practice (Clarke et al. 2021). In addition, Golge et al. (2024) reported that the overall implementation of EBP among nurses was relatively low, with only 54.5% using it consistently. Furthermore, most respondents indicated that they had not used a systematic review report (61.0%) or critically appraised evidence from a research study (60.3%) in the previous 8 weeks. However, according to literature, several barriers hinder nurses' attitudes towards EBP, including heavy workload, lack of time and insufficient support from colleagues or managers, which limit nurses' engagement with EBP (Clarke et al. 2021). Leach and Veziari (2022) highlighted limited access to information technologies, a lack of confidence in evaluating research, and challenges in understanding statistics and scientific terminology. Mathieson et al. (2019) further emphasized the lack of knowledge and skills required to transfer research findings into practice and inadequate foreign language proficiency to follow current scientific developments. Additionally, inadequate collaboration in academic and clinical fields in the nursing profession may cause nurses to fail to reflect on scientific and EBPs in patient care (Alatawi et al. 2020). Therefore, it is essential to determine the factors affecting nurses' EBP competencies and CDM skills.

This study used a mixed‐methods design to determine nurses' EBP competencies and CDM levels and the factors influencing these competencies. EBP competencies, which encompass the knowledge, skills and attitudes required to integrate evidence into practice, are critical because they enable nurses to deliver safe, effective and standardized care, reduce clinical errors and ensure that interventions are based on the best available evidence (Clarke et al. 2021; Golge et al. 2024). CDM processes, in turn, are central to evaluating patient needs, comparing alternative interventions, anticipating outcomes and making timely and accurate clinical judgments (Standing 2020). Combined, these competencies directly contribute to patient safety, quality of care and the professional development of nurses (Watkins 2020; WHO 2017). However, studies that examine the relationship between nurses' EBP competencies and CDM processes in depth remain limited in the literature, which creates a significant gap in understanding how these interrelated domains can be developed and supported. This study addresses this gap by providing evidence on how EBP competencies are associated with CDM levels in nursing and by offering insights into how working conditions, professional experience and organizational structures shape these competencies. These findings may serve as useful guidance for educators, administrators and policymakers seeking to support the wider integration of EBP in clinical settings.

## 3 The Study

### 3.1 Design

This study utilized a convergent parallel design, a mixed‐method approach, to explore factors affecting nurses' competencies in EBPs and CDM. Mixed‐method research combines qualitative and quantitative methods for a comprehensive analysis (Yıldırım and Şimşek 2013). In this design, qualitative and quantitative data are collected simultaneously, analysed separately and integrated during interpretation (Clark and Ivankova 2015). The quantitative phase included a questionnaire measuring EBP competencies and CDM levels, while the qualitative phase involved focus group interviews based on van Manen's (1990) interpretative phenomenology model.

### 3.2 Aim and Sub‐Aims

The study aimed to examine the competencies of nurses regarding EBP and their CDM processes. The sub‐aims of the study are as follows:

1. To determine the competency levels of nurses towards EBPs and the CDM.
2. To identify the factors influencing nurses' competency levels towards EBPs and CDM.
3. To examine the relationship between nurses' competency levels towards EBPs and their CDM.
4. To explore nurses' attitudes, observations and experiences related to EBPs and CDM.

### 3.3 Participants and Setting

The study population consisted of 243,565 nurses working in the Ministry of Health, universities and private institutions in Türkiye (MoH 2023). The quantitative part sample size of the study was calculated using Cochran's (1977) method for known populations, yielding a required sample of 385 with a 95% confidence interval and 5% margin of error. The study was completed with 387 participants. A post hoc power analysis conducted with G*Power 3.1.9.6 confirmed a 96% power and an effect size of 0.67 (*f* ^2^), indicating adequate sample size. The qualitative part of the study sample size was determined according to reaching data saturation and consisted of 20 nurses who voluntarily agreed to participate. For both parts, the inclusion criteria required being an actively working registered nurse; retired nurses, those not currently working in clinical practice and nurses who left their jobs during the research process were excluded. Participants were recruited through institutional mailing lists and online survey distribution via Qualtrics, and all participation was voluntary without any form of compensation. Most of the study settings were in urban areas.

### 3.4 Instruments

The Descriptive Information Form for Nurses, Evidence‐based Practice Evaluation Competence Questionnaire (EBP‐COQ) Scale, The Clinical Decision‐Making in Nursing Scale (CDMNS), and Semi‐structured Interview Form for Nurses were used to collect the study data.

#### 3.4.1 Descriptive Information Form for Nurses

This form was prepared based on the literature (Alatawi et al. 2020; Clarke et al. 2021) and consisted of 16 questions regarding socio‐demographic and professional information of nurses.

#### 3.4.2 The Evidence‐Based Practice Evaluation Competence Questionnaire (EBP‐COQ)

The EBP‐COQ scale was developed by Ruzafa‐Martinez et al. (2013) and assesses nurses' and nursing students' competence in evidence‐based practices (EBPs). The Turkish validity and reliability were conducted by Yildiz and Güngörmüş (2016). The scale comprises three sub‐dimensions (attitude: items 1–13, skill: items 14–16, and knowledge: items 20–25) and 25 items in total and is rated on a five‐point Likert scale. Scores ranged from 25 to 125, with higher scores indicating greater self‐perceived competence, knowledge, skills and positive attitudes towards EBPs. Negative items were reverse‐coded. The Cronbach's Alpha value was 0.88 in the original study and 0.82 in the Turkish adaptation. In this study, the scale's Cronbach's Alpha was 0.91, indicating high reliability.

#### 3.4.3 The Clinical Decision‐Making in Nursing Scale (CDMNS)

The CDMNS was developed by Jenkins (1985) to assess nursing students' decision‐making levels, though it can also be used for clinical nurses (Jenkins 2001). The Turkish validity and reliability were established by Durmaz Edeer and Sarıkaya (2015). The scale comprises 40 items across four subscales: ‘investigating options and ideas’, ‘investigating goals and values’, ‘evaluating results’ and ‘investigating information and adopting new information impartially’, each containing 10 items. The scale uses a five‐point Likert format, with 22 positive and 18 reverse‐scored negative items. Total scores ranged from 40 to 200, with subscales between 10 and 50, and higher scores indicating better decision‐making perceptions. In Jenkins' (1985) original study, Cronbach's Alpha was 0.83, while Durmaz Edeer and Sarıkaya (2015) reported 0.78. In this study, the Cronbach's Alpha was 0.79, indicating good reliability.

#### 3.4.4 Semi‐Structured Interview Form for Nurses

This form was prepared in line with the literature and was used in the focus group interviews. The form consists of 10 open‐ended questions regarding nurses' opinions, experiences, and suggestions regarding EBPs and CDM to organize and provide detailed data on the subject and guide the researcher during the interview (Jun et al. 2016; Golge et al. 2024). The guiding questions addressed multiple aspects of EBP, including usage, perceived competencies, impacts, barriers and facilitators. Specifically, the interview form included the following questions:

- What comes to your mind when you hear the term ‘EBPs’?
- Can you provide examples of EBPs that you use or are familiar with in patient care processes?
- What are your opinions and experiences regarding nurses' competence and ability to use EBPs?
- Which methods do you use when making clinical decisions during patient care processes? Please share your opinions and experiences.
- What are the positive or negative effects of EBPs on the nursing profession?
- What are the positive or negative effects of EBPs on patient outcomes?
- What factors facilitate the use of EBPs by nurses? Please share your opinions and experiences.
- What factors hinder nurses from using EBPs? Please explain.
- Does your institution use any methods or programs to support the use of EBPs by nurses? If yes, could you describe your experiences with them?
- In your opinion, what could be done to increase nurses' competence and attitudes towards EBPs?

These questions were designed to capture nurses' perspectives on both the practical and contextual dimensions of implementing EBPs in clinical decision‐making, while also allowing for the identification of institutional support and challenges.

### 3.5 Data Collection

A pilot study was conducted with five nurses outside the sample group to refine the data collection tools by addressing unclear or incomplete items. For the quantitative part, data were collected through the ‘Descriptive Information Form for Nurses’, ‘EBP‐COQ’ and ‘CDMNS’ using the Qualtrics online survey platform. Participants received the survey link, along with weekly reminder emails. Informed consent was obtained online, and participants completed the survey in approximately 10–15 min. In the qualitative part, focus group interviews were conducted with 20 nurses in Türkiye, working in both state and foundation/private hospitals, predominantly located in urban areas. Informed consent was obtained, and four face‐to‐face sessions, each with five participants, were moderated by trained PhD female researchers (S.S. and P.K.). The ‘Semi‐structured Interview Form for Nurses’ guided the discussions, which were audio‐recorded and lasted around 45 min each. Data collection continued until saturation was reached, with additional probing questions used to clarify key themes. Participants' identities were anonymized, coded as P1, P2, P3, etc.

### 3.6 Data Analysis

The study was reported in accordance with the Good Reporting of a Mixed‐Methods Study (GRAMMS) guidelines (O'Cathain et al. 2008), which outline standards for integrating quantitative and qualitative approaches in mixed‐methods research. Quantitative analysis was performed using IBM SPSS 27.0 (IBM Corp., Armonk, NY, USA). Normality of continuous variables was assessed through skewness and kurtosis values (−1.5 to +1.5). Reliability was evaluated with Cronbach's Alpha. Frequencies (*n*, %) and mean ± standard deviation were used for categorical and continuous variables, respectively. Independent *t*‐tests and one‐way ANOVA (with Scheffe's post hoc test) compared groups based on sociodemographic and professional characteristics such as age, marital status, education level, clinical position, workplace type, working hours and shift status. Pearson's correlation assessed relationships between EBP‐COQ and CDMNS scores. Stepwise multiple linear regression was conducted to examine the effect of independent variables (education level, clinical position, workplace type, professional experience, foreign language knowledge, training in EBP, following scientific publications and participation in research activities) on dependent variables (EBP‐COQ and CDMNS scores). Statistical significance was set at *p* < 0.05.

Qualitative data were analysed using content analysis, following the structured protocol of Elo and Kyngäs (2008), which involves three systematic phases: preparation (immersing in the data and defining the unit of analysis), organizing (open coding, creating categories and grouping codes into higher‐order categories) and reporting (describing categories and themes to capture the meaning of the data). Two researchers (S.S. and P.K.), experienced in qualitative research, independently coded the data line by line, categorized codes and abstracted themes. Themes and sub‐themes were finalized after joint review and consensus to ensure trustworthiness. The qualitative analysis was supported by MAXQDA software.

Quantitative and qualitative findings were integrated through a side‐by‐side comparison and presented in a joint display table. Convergence was observed where high EBP and CDM scores aligned with qualitative reports of applying evidence‐based protocols. Divergence appeared when quantitative results showed lower scores among nurses with long working hours or inpatient roles, while qualitative data emphasized workload and resource limitations as barriers. Complementarity emerged as quantitative predictors such as education, research experience, and foreign language skills were enriched by qualitative insights on critical thinking, professional growth and organizational culture. Together, these findings provided a comprehensive understanding of nurses' engagement with EBP and CDM.

### 3.7 Rigour and Trustworthiness of Qualitative Analysis

Credibility, transferability, dependability and conformability were maintained to assure the reliability of the qualitative data analysis (Johnson et al. 2020; Lincoln and Guba 1986). To manage group dynamics during focus group interviews and prevent dominance bias, the moderator encouraged participation from all members, invited quieter participants to contribute, and ensured that no single voice dominated the discussion. The key points were summarized at the end of each interview. This process also served as a form of member checking, allowing participants to confirm, expand, or correct the researchers' interpretations. All participants were invited to add new information or clarify their answers post‐interview. In addition, credibility was ensured by the first author's review of the written expressions regarding whether there is incomprehensiveness in the collected data. The expression did not convey any ambiguity. Transferability was established through a delicate and detailed explanation in the methodology section. All participants participated in this study voluntarily. Conformability was maintained by conducting the interviews with a single researcher. Dependability and analytical rigor were strengthened through intercoder reliability measures: two researchers independently coded the transcripts, compared the coding outcomes, and resolved discrepancies through discussion until consensus was reached. Sample quotes were taken directly from interviewee reports.

### 3.8 Ethical Considerations

Ethics committee permission was obtained from the Koç University Ethics Committee on Human Research for this study (2024.169.IRB3.076). Permissions for using scales were received by e‐mail from Yıldız, who performed the validity and reliability of EBP‐COQ, and Durmaz Edeer, who performed the validity and reliability of the CDMNS. For the quantitative phase, participants received detailed information about the study on the first page of the online survey, and informed consent was obtained electronically before participation. For the qualitative phase, nurses were provided with detailed information about the research prior to the focus group interviews, and written informed consent was obtained. Participants were also informed that the interviews would be audio‐recorded and their identifying information would not be shared. It was explained that only the research team could access the recorded data, which would be used solely for research purposes.

## 4 Results

### 4.1 Quantitative Results

#### 4.1.1 Descriptive Characteristics of the Nurses

Data from a total of 387 nurses were analysed in the study. The mean age of the nurses was 28.64 ± 4.45 years, 72.6% were female and 87.4% had a bachelor's degree or higher. The nurses' mean duration of professional experience was 5.78 ± 4.73 years, and the mean duration of working in the institution was 4.72 ± 4.013 years. Of the nurses, 74.9% worked as clinical nurses, 58.7% worked in private hospitals, and 80.4% worked in a shift system. It was found that 62% of the nurses worked 48 h or more per week, 42.4% had a foreign language, 19.1% had received training related to EBP, 52.5% followed scientific publications, 48.8% participated in professional, scientific activities and 27.6% were involved in research planning and implementation (Table 1).

**TABLE 1:** Descriptive characteristics of the nurses.

Variables (*N* = 387)	Category	*n* (%)
Age (years)	Mean ± SD	28.64 ± 4.45
Age group	< 28	174 (45)
	≥ 28	213 (55)
Gender	Man	106 (27.4)
	Female	281 (72.6)
Marital status	Married	215 (55.6)
	Single	172 (44.4)
Level of education	Health vocational high school	22 (5.7)
	Associate degree	27 (7)
	Licence	282 (72.9)
	Postgraduate	56 (14.5)
Institution	State hospital	160 (41.3)
	Foundation/Private hospital	227 (58.7)
Position	Clinical Nurse	290 (74.9)
	Specialty Nurse	42 (10.9)
	Manager	55 (14.2)
Duration of professional experience (years)	Mean ± SD	5.78 ± 4.73
Duration of professional experience group	< 6	212 (54.8)
	≥ 6	175 (45.2)
Duration of institutional experience (years)	Mean ± SD	4.72 ± 4.013
Duration of the institutional experience group	< 5	219 (56.6)
	≥ 5	168 (43.4)
Working unit	Inpatient units	229 (59.2)
	Outpatient clinics	18 (4.7)
	Intensive care units	96 (24.8)
	Surgical and Interventional Units	12 (3.1)
	Emergency department	20 (5.2)
	Nursing administration	12 (3.1)
Working system	Fixed schedule	76 (19.6)
	Shift system	311 (80.4)
Weekly working hours	40 h	20 (5.2)
	48 h	127 (32.8)
	≥ 48 h	240 (62)
Second language	Yes	164 (42.4)
	No	223 (57.6)
Receiving EBP education	Yes	74 (19.1)
	No	313 (80.9)
Following professional scientific publications	Yes	203 (52.5)
	No	184 (47.5)
Participation in a professional scientific event	Yes	189 (48.8)
	No	198 (51.2)
Experience in research planning and implementation	Yes	107 (27.6)
	No	280 (72.4)

Abbreviation: SD, Standard deviation.

#### 4.1.2 Nurses' EBP‐COQ and CDMNS Mean Scores

The mean EBP‐COQ total score of the nurses was 91.34 (SD = 12.78), corresponding to approximately 75% of the maximum possible score, indicating a generally high level of EBP competence. Subscale analyses showed mean scores of 54.80 (SD = 6.03) for attitudes (reflecting perceptions and values towards EBP), 18.93 (SD = 3.96) for skills (assessing practical ability to apply EBP) and 17.61 (SD = 4.34) for knowledge (evaluating theoretical understanding of EBP principles). When expressed relative to the maximum possible values, nurses' competence levels were highest for attitudes (about 84%), while knowledge (59%) and skills (63%) were comparatively lower. This suggested that while nurses maintain positive orientations towards EBP, their theoretical and practical capacities remain more limited.

The mean CDMNS total score was 147.5 (SD = 11.96), equivalent to approximately 80% of the maximum possible score, suggesting a high level of clinical decision‐making skills. Sub‐dimension mean scores were 38.92 (SD = 4.22) for investigating options and ideas (generating alternatives), 35.56 (SD = 3.09) for investigating goals and values (clarifying priorities), 39.17 (SD = 3.85) for evaluating results (assessing decision outcomes) and 33.84 (SD = 3.56) for searching for and adopting new information impartially (integrating new evidence without bias). The results indicated that nurses were particularly strong in evaluating the results of their decisions, while they showed relative weakness in incorporating new information, reflecting a need for greater support in analytical and evidence‐seeking skills (Table 2).

**TABLE 2:** Descriptive statistics and correlations of EBP‐COQ and CDMNS scores among nurses (*N* = 387).

No	Scales	Mean ± SD	Range	1	2	3	4	5	6	7	8
1	EBP‐COQ‐Attitude	54.80 ± 6.03	32–65	N/A							
2	EBP‐COQ‐Skill	18.93 ± 3.96	7–28	0.667*							
3	EBP‐COQ‐Knowledge	17.61 ± 4.34	7–29	0.651*	0.771*						
4	EBP‐COQ‐Total	91.34 ± 12.78	49–121	0.900*	0.886*	0.886*					
5	CDMNS‐O&I	38.92 ± 4.22	24–50	0.700*	0.544*	0.578*	0.695*				
6	CDMNS‐G&V	35.56 ± 3.09	26–45	0.574*	0.481*	0.453*	0.574*	0.615*			
7	CDMNS‐ER	39.17 ± 3.85	27–49	0.590*	0.449*	0.451*	0.571*	0.682*	0.616*		
8	CDMNS‐SI&ANI	33.84 ± 3.56	25–45	0.423*	0.433*	0.424*	0.478*	0.464*	0.398*	0.464*	
9	CDMNS‐Total	147.5 ± 11.96	109–184	0.711*	0.589*	0.592*	0.719*	0.869*	0.792*	0.859*	0.713*

*Note:* *p < 0.001‐Spearman correlation test; Clinical Decision‐Making in Nursing Scale (CDMNS), investigating options and ideas (O&I), investigating goals and values (G&V), evaluating the results (ER), searching for information and adopting new information impartially (SI&ANI), Evidence‐based Practice Evaluation Competence Questionnaire (EBP‐COQ).

Abbreviation: N/A, not available.

#### 4.1.3 Comparison of the Nurses' Mean EBP‐COQ and CDMNS Scores and the Descriptive Characteristics

Nurses aged 28 years and over [(EBP‐COQ, *t* = 2.679; *p* = 0.008) and (CDMNS, *t* = 3.062; *p* = 0.002)] and married [(EBP‐COQ, *t* = 2.507; *p* = 0.013) and (CDMNS, *t* = 3.649; *p* < 0.001)] had statistically significantly higher levels of competence towards EBPs and CDM. As the level of education increased, nurses' levels of competence towards EBPs (EBP‐COQ, *F* = 152.607; *p* < 0.001) and CDM (CDMNS, *F* = 95.623; *p* < 0.001) also increased statistically significantly. Compared to other positions, nurses working as clinical nurses had significantly lower levels of competence in EBPs (EBP‐COQ, *F* = 21.458; *p* < 0.001) and CDM (CDMNS, *F* = 15.119; *p* < 0.001).

There was a statistically significant difference in the mean EBP‐COQ and CDMNS scores of nurses according to the unit they worked in. Nurses working in inpatient units had statistically significantly lower levels of competence towards EBPs (EBP‐COQ, *F* = 7.352; *p* < 0.001) and CDM (CDMNS, *F* = 3.156; *p* = 0.044). The CDM level of nurses with 6 or more years of professional experience (CDMNS, *t* = 2.741; *p* = 0.006) or 5 or more years of experience in their last institution (CDMNS, *t* = 2.451; *p* = 0.015) was statistically significantly higher.

The competence and CDM levels towards EBPs of nurses who work in shift system [(EBP‐COQ, *t* = 5.221; *p* < 0.001) and (CDMNS, *t* = 3.895; *p* < 0.001)] or who work 48 h or more per week [(EBP‐COQ, *F* = 16.979; *p* < 0.001) and (CDMNS, *F* = 4.430; *p* = 0.013)] are statistically significantly lower. It was found that nurses who spoke a foreign language, received training in EBP‐COQ, followed professional, scientific publications, participated in a professional, scientific activity, and had previously participated in research planning‐implementation had statistically significantly higher levels of competence and CDM towards EBPs [(EBP‐COQ, *t* = 3.759 to 15.118; *p* < 0.001) and (CDMNS, *t* = 2.690 to 7.103; *p* < 0.01 and *p* < 0.001)] (Table 3).

**TABLE 3:** Comparison of nurses' descriptive characteristics and EBP‐COQ and CDMNS scores (*N* = 387).

Variables	Category	*n*	EBP‐COQ		CDMNS	
			Mean ± SD	Statistical analysis	Mean ± SD	Statistical analysis
Age group	< 28	174	89.38 ± 14.30	*t* = 2.679	145.39 ± 13.66	*t* = 3.062
	≥ 28	213	92.93 ± 11.16	*p* = 0.008*	149.20 ± 10.08	*p* = 0.002*
Gender	Man	106	89.69 ± 13.52	*t* = 1.561	147.18 ± 12.12	*t* = 0.312
	Woman	281	91.96 ± 12.45	*p* = 0.119	147.60 ± 11.92	*p* = 0.755
Marital status	Married	215	92.83 ± 10.72	*t* = 2.507	149.50 ± 10.24	*t* = 3.649
	Single	172	89.47 ± 14.78	*p* = 0.013*	144.98 ± 13.42	*p* < 0.001*
Level of education	HVHS^1^	22	64.32 ± 14.01	*F* = 152.607	122.41 ± 12.69	*F* = 95.623
	Associate Degree^2^	27	82.33 ± 10.98	*p* < 0.001*	135.33 ± 10.71	*p* < 0.001*
	License^3^	282	90.95 ± 7.75	diff **4 > 3 > 2 > 1	148.65 ± 8.02	diff **4 > 3 > 2 > 1
	Postgraduate^4^	56	108.25 ± 9.11		157.34 ± 11.40	
Institution	State hospital	160	91.72 ± 13.84	*t* = 0.494	147.28 ± 12.28	*t* = 0.294
	Foundation/Private hospital	227	91.07 ± 11.99	*p* = 0.621	147.64 ± 11.75	*p* = 0.769
Position	Clinic Nurse^1^	290	89.03 ± 13.04	*F* = 21.458	145.63 ± 12.27	*F* = 15.119
	Specialty Nurse^2^	42	96.55 ± 9.17	*p* < 0.001*	153.88 ± 7.55	*p* < 0.001*
	Manager^3^	55	99.49 ± 8.68	diff **1 < 2, 3	152.38 ± 9.96	diff **1 < 2, 3
Duration of professional experience	< 6	212	90.21 ± 13.46	*t* = 1.919	146.01 ± 12.69	*t* = 2.741
	≥ 6	175	92.70 ± 11.79	*p* = 0.056	149.28 ± 10.78	*p* = 0.006
Duration of institutional experience	< 5	219	90.27 ± 13.40	*t* = 1.881	146.19 ± 12.45	*t* = 2.451
	≥ 5	168	92.73 ± 11.81	*p* = 0.061	149.18 ± 11.10	*p* = 0.015
Working unit	Inpatient units^1^	229	89.42 ± 13.75	*F* = 7.352	146.39 ± 13.19	*F* = 3.156
	Intensive care units^2^	96	93.05 ± 9.92	*p* < 0.001*	148.15 ± 9.61	*p* = 0.044*
	Other units^3^	62	95.74 ± 11.64	diff **1 < 3	150.53 ± 9.81	diff **1 < 3
Working system	Fixed schedule	76	97.97 ± 9.76	*t* = 5.221	151.61 ± 9.74	*t* = 3.895
	Shift system	311	89.71 ± 12.91	*p* < 0.001*	146.48 ± 12.25	*p* < 0.001*
Weekly working hours	40 h^1^	20	97.25 ± 10.90	*F* = 16.979	149.30 ± 13.58	*F* = 4.430
	48 h^2^	127	95.76 ± 11.97	*p* < 0.001*	149.85 ± 12.03	*p* = 0.013
	≥ 48 h^3^	240	88.50 ± 12.54	diff **3 < 1, 2	146.09 ± 11.61	diff **3 < 2
Second language	No	223	89.22 ± 11.58	*t* = 3.759	145.61 ± 11.45	*t* = 3.617
	Yes	164	94.21 ± 13.77	*p* < 0.001*	150.04 ± 12.20	*p* < 0.001*
Receiving EBP education	No	313	90.00 ± 12.44	*t* = 4.255	146.70 ± 12.12	*t* = 2.690
	Yes	74	96.97 ± 12.73	*p* < 0.001*	150.82 ± 10.71	*p* = 0.007*
Following professional scientific publications	Yes	203	95.23 ± 14.57	*t* = 6.802	149.51 ± 12.90	*t* = 3.586
	No	184	87.04 ± 8.63	*p* < 0.001*	145.26 ± 10.41	*p* < 0.001*
Participation in a professional scientific event	Yes	189	96.29 ± 13.25	*t* = 8.000	150.60 ± 11.67	*t* = 5.170
	No	198	86.61 ± 10.31	*p* < 0.001*	144.52 ± 11.49	*p* < 0.001*
Experience in research planning and implementation	Yes	107	103.93 ± 10.14	*t* = 15.118	154.07 ± 11.64	*t* = 7.103
	No	280	86.52 ± 10.13	*p* < 0.001*	144.98 ± 11.11	*p* < 0.001*

*Note:* *p < 0.05; *t*, independent sample *t*‐test; *F*, One‐way ANOVA test; **Scheffe post hoc test.

Abbreviations: CDMNS, Clinical Decision‐Making in Nursing Scale; EBP‐COQ, Evidence‐based Practice Evaluation Competence Questionnaire; HVHS, Health Vocational High School; SD, Standard deviation.

#### 4.1.4 Factors Associated With EBP‐COQ and CDMNS Scores

Multiple linear regression analysis was performed with the stepwise method to determine the factors affecting the EBP‐COQ level of nurses by using the factors found significant in the univariate analysis. With the regression model (model 5), it was found that the independent variables explained approximately 64.3% (adjusted *R* ^2^ = 0.643) of the change in EBP‐COQ level. Accordingly, increasing educational level (*β* = 0.554; *p* < 0.001), experience in research planning and implementation (*β* = 0.287; *p* < 0.001) and following professional scientific publications (*β* = 0.092; *p* = 0.009) positively associated with nurses' level of competence towards EBPs; in clinical nurse position (*β* = −0.081; *p* = 0.020) and working in inpatient units (*β* = −0.083; *p* = 0.014) negatively associated with nurses' level of competence towards EBPs (Table 4). A one‐unit increase in education level was associated with a 0.55‐point increase in the EBP competence score, while research planning and implementation experience contributed approximately 0.29 points. Following scientific publications was associated with a 0.09‐point increase. In contrast, being in a clinical nurse position or working in inpatient units was associated with decreases of about 0.08 points in the EBP competence score.

**TABLE 4:** Factors associated with nurses' EBP‐COQ and CDMNS scores (*N* = 387).

Dependent variable: EBP‐COQ								
Model	*R*	*R* ^2^	Adjusted *R* ^2^	SE of estimation	Change statistics		Sig. of change in *F*	DW
					*R* ^2^	*F*		
1	0.726	0.527	0.526	8.798	0.527	429.029	< 0.001	
2	0.789	0.623	0.621	7.867	0.096	97.571	< 0.001	
3	0.798	0.636	0.634	7.734	0.014	14.311	< 0.001	
4	0.801	0.642	0.638	7.686	0.005	5.791	0.017	
5	0.805	0.648	0.643	7.635	0.006	6.087	0.014	1.709

Model 5	Unstandardized coefficients		Standardized coefficients	*t*	*p*	Collinearity statistics		
	*B*	SE	*β*			Tolerance	VIF	
(Constant)	59.320	2.198		26.987	< 0.001			
Level of education	10.661	0.668	0.554	15.970	< 0.001	0.768	1.303	
Experience in research planning and implementation	8.199	1.116	0.287	7.349	< 0.001	0.605	1.653	
Working as clinic nurse	−2.383	1.018	−0.081	−2.342	0.020	0.774	1.291	
Following professional scientific publications	2.350	0.892	0.092	2.634	0.009	0.759	1.318	
Working in inpatient units	−2.142	0.868	−0.083	−2.467	0.014	0.827	1.209	

Dependent variable: CDMNS								
Model	*R*	*R* ^2^	Adjusted *R* ^2^	SE of estimation	Change statistics		Sig. of change in *F*	DW
					*R* ^2^	*F*		
1	0.719	0.517	0.516	8.321	0.517	412.442	< 0.001	
2	0.742	0.550	0.548	8.044	0.033	27.961	< 0.001	
3	0.753	0.567	0.564	7.899	0.017	15.183	< 0.001	
4	0.756	0.572	0.567	7.868	0.004	3.994	0.046	1.538

Model 4	Unstandardized coefficients		Standardized coefficients	*t*	*p*	Collinearity statistics		
	*B*	SE	*β*			Tolerance	VIF	
(Constant)	74.775	5.238		14.277	< 0.001			
EBP‐COQ	0.564	0.052	0.603	10.858	< 0.001	0.364	2.751	
Level of education	4.950	0.878	0.275	5.637	< 0.001	0.471	2.123	
Experience in research planning and implementation	4.620	1.134	0.173	4.074	< 0.001	0.622	1.608	
Working as clinic nurse	−1.951	0.976	−0.071	−1.998	0.046	0.894	1.119	

Abbreviations: CDMNS, Clinical Decision‐Making in Nursing Scale; DW, Durbin Watson; EBP‐COQ, Evidence‐based Practice Evaluation Competence Questionnaire; SE, Standard error; VIF, Variance Inflation Factor.

Using the factors found to be significant in the univariate analysis, multiple linear regression analysis was performed with the stepwise method to determine the factors associated with the CDMNS level of nurses. With the final regression model (model 4), the independent variables explained approximately 56.7% of the change in the CDMNS level (adjusted *R* ^2^ = 0.567) Accordingly, increasing education (*β* = 0.275; *p* < 0.001) and EBP‐COQ level (*β* = 0.603; *p* < 0.001) and experience in research planning and implementation (*β* = 0.173; *p* < 0.001) positively affected nurses' CDM, whereas working in a clinical nurse position (*β* = −0.071; *p* = 0.046) negatively affected nurses' CDM (Table 4). An additional unit in education level was associated with a 0.27‐point increase in CDM scores, while higher EBP competence accounted for the largest contribution with an increase of 0.60 points. Research experience was associated with an increase of 0.17 points, whereas being in a clinical nurse position was associated with a decrease of 0.07 points in CDM scores.

#### 4.1.5 The Correlation Between EBP‐COQ and CDMNS Scores

There was a statistically significant and highly positive relationship between EBP‐COQ and CDMNS total scores of nurses (*r* = 0.719; *p* < 0.001). The relationship between the total and subscale scores of the scales is given in Table 2.

### 4.2 Qualitative Results

**FIGURE 1:**
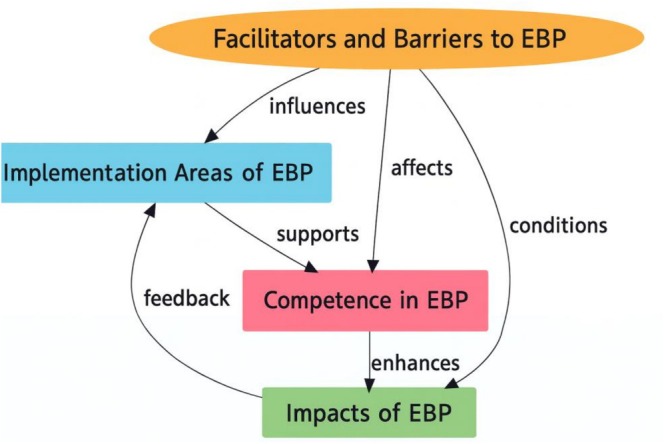
Thematic framework of evidence‐based practice: Relationships between themes.

Nurses who participated in the qualitative part of the study, 75% were female, 80% were undergraduate graduates, 54% were single, 70% worked in inpatient services and 65% worked in private hospitals. The mean age of the nurses was 25.34 years (SD ±3.45), and the mean duration of professional experience was 4.75 years (SD ±3.74). All the nurses were working with a shift system, and 75% of them stated that they had not received training on EBPs before. According to the results of the descriptive analysis conducted after the focus group interviews with the nurses, four main themes and 10 sub‐themes were identified in total, including ‘Implementation Areas of EBP’, ‘Competence in EBP’, ‘Impacts of EBP’ and ‘Facilitators and Barriers to EBP’ (Table 5). To provide a clearer overview of how these themes are interconnected, a thematic framework is presented in Figure 1. The framework illustrates that Implementation Areas of EBP provide the basis for applying EBPs. These areas strengthen Competence in EBP, shaped by individual factors such as critical thinking, education, professional experience and language skills, as well as organizational resources including training and institutional support. Higher competence enhances the Impacts of EBP, which include standardized care, professional development, patient safety and quality of care. Facilitators and Barriers to EBP influence all themes, with supportive conditions promoting competence and impact, while constraints such as workload, staffing shortages, limited resources and low motivation limit the effective use of EBPs.

**TABLE 5:** Main Themes and Subthemes Regarding Nurses' Opinions and Experiences on Evidence‐Based Practices (*n*: 20).

Themes	Subthemes
Implementation Areas of EBP	Implementation in nursing care processes and clinical decision‐making Integration of managerial processes
Competence in EBP	Individual factors Professional factors Organizational factors
Impacts of EBP	Integrating scientific decision‐making with standard nursing practices Enhancing patient care quality and safety Improving nurses' work environments and professional development
Facilitators and Barriers to EBP	Facilitators: Education, supportive working conditions, technology, and organizational resources Barriers: Patient attitudes, individual and organizational challenges, and equipment limitations

#### 4.2.1 Theme 1. Implementation Areas of EBP

Two sub‐themes were identified within this theme: implementation in nursing care processes and clinical decision‐making and integration of managerial processes. Most nurses emphasized that they integrated EBPs into basic nursing practices and patient care, such as following institutional protocols and checklists to ensure safe and standardized procedures. For example, one nurse described: ‘In patient care, we generally follow the protocols, checklists, etc., in our institution. For example, when the patient's port catheter needle is to be changed, we definitely perform this procedure in an evidence‐based manner; we follow the protocols for this practice’ (Group 2, P7). In addition, several participants highlighted the use of EBPs in managerial processes, such as planning, supervising and evaluating nursing care within their units. Nurses also indicated that their clinical decision‐making was guided by evidence‐based approaches, supporting both patient care and managerial responsibilities. *As one participant noted*, ‘As a charge nurse, I often have to organize the unit schedule and supervise daily tasks. I try to use evidence‐based guidelines for staffing and care planning, not just my own experience’ (Group 1, P3).

#### 4.2.2 Theme 2. Competence in EBP

Three sub‐themes were identified within this theme: individual factors, professional factors and organizational factors. Nurses explained that individual characteristics, such as having critical thinking skills and being able to follow foreign‐language resources, contributed positively to their EBP competence. Professional factors, including undergraduate or graduate education and prior clinical experience, were also emphasized as important enablers. As one participant reflected, ‘…there were times when we gained competence in some practices through trial and error when we first started working, but as we gain experience and accelerate in our work, we start to research how to do this practice better, and that is when we start to make evidence‐based scientific decisions’ (Group 4, P18). In addition, organizational factors were highlighted as critical, particularly the availability of resources, the presence of a supportive institutional culture, and access to structured training. Nurses frequently noted that without this organizational support, their individual and professional capacities alone were insufficient to sustain evidence‐based practice. As one nurse expressed, ‘Even if I want to use evidence‐based practice, without enough resources and support from the hospital, it is very difficult to sustain it’ (Group 2, P8).

#### 4.2.3 Theme 3. Impacts of EBP

Three sub‐themes were identified within this theme: integrating scientific decision‐making with standard nursing practices, enhancing patient care quality and safety, and improving nurses' work environments and professional development. Many nurses emphasized that EBPs facilitated clinical decision‐making grounded in science, which in turn supported the standardization of nursing practices and strengthened their professional knowledge and skills. Several participants highlighted that EBPs contributed to the quality and safety of patient care, particularly by reducing risks such as infections and by promoting patients' sense of security. As one nurse explained, ‘…it definitely has a positive effect. EBPs improve the quality of patient care, reduce infection risks, and positively affect patients' psychological well‐being. For example, performing procedures such as double‐checking or identity verification in the presence of the patient makes them feel safe’ (Group 1, P5). In addition, some participants noted that EBPs positively influenced their professional development and image by fostering more supportive and structured work environments. As one nurse emphasized, ‘Using evidence‐based approaches gives me pride in my work and shows that nursing is a scientific profession, not just routine tasks’ (Group 3, P14).

#### 4.2.4 Theme 4. Facilitators and Barriers to EBP

Two sub‐themes were identified within this theme: facilitators and barriers. Nurses reported that several organizational and educational factors acted as facilitators, including supportive working conditions, participation in scientific activities, access to postgraduate programs and training, as well as technological infrastructure and institutional resources. These enablers were described as crucial in helping nurses adopt EBPs more effectively in daily practice. As one nurse explained, ‘When the hospital provides training and encourages us to attend conferences, it becomes much easier to apply EBPs in daily care’ (Group 1, P2). Conversely, participants highlighted multiple barriers at both individual and organizational levels. Negative organizational conditions such as heavy workload, insufficient staffing, and lack of institutional resources were frequently cited as major obstacles. Some nurses also noted individual barriers, including perceiving EBPs as additional workload, low motivation, limited knowledge and negative attitudes. The challenge of patient‐related factors and the lack of adequate equipment further hindered EBP use. As one nurse explained, ‘Of course, we want to provide evidence‐based care, but we are so busy that there are not enough nurses, and it is difficult to keep up with every patient. I mean, we do not have the time to open it at that moment and see how this practice should be the most up‐to‐date and the most accurate. In addition, it is difficult to access this evidence; there are no information booklets, etc.…’ (Group 3, P13).

### 4.3 Integrated Findings

The integration of quantitative and qualitative results showed that nurses demonstrated high levels of competence in both EBP and CDM. The high mean EBP‐COQ and CDMNS scores were consistent with qualitative reports indicating that nurses frequently applied institutional protocols and guidelines in patient care and managerial processes. Quantitative analyses identified education, research experience, and the following scientific publications as significant positive predictors of EBP competence. This was supported by qualitative findings, in which nurses described that higher education, critical thinking, and involvement in research activities enhanced their competence in using EBPs. Lower EBP competence among clinical nurses and those working in inpatient units was also identified in the quantitative results. Qualitative data explained these findings by emphasizing barriers such as workload, insufficient staffing, and limited resources that constrained the use of EBPs in practice. Education, EBP competence, and research experience were found to be positive predictors of CDM, while the clinical nurse position emerged as a negative predictor. Qualitative findings confirmed these results by showing that EBPs facilitated decision‐making, improved standardization of care, and enhanced patient safety, whereas nurses in clinical positions described limited resources and workload as constraints on applying evidence‐based decision‐making (Table 6).

**TABLE 6:** Integration of Quantitative and Qualitative Findings on Factors Influencing Evidence‐Based Practice and Clinical Decision‐Making.

Quantitative findings	Qualitative findings	Integrated interpretation
Nurses demonstrated high competence levels in both EBP and CDM domains (EBP‐COQ total = 91.34; CDMNS total = 147.5)	Nurses frequently reported applying protocols and guidelines in daily care and managerial processes. ‘We definitely perform this procedure in an evidence‐based manner; we follow the protocols…’ (G2, P7)	The high quantitative scores are substantiated by qualitative accounts showing that nurses actively integrate EBPs into their routines. This reinforces that self‐reported competence aligns with actual practice behaviours and also demonstrates that stronger EBP competence directly contributes to more consistent and evidence‐informed clinical decision‐making
Higher education (*β* = 0.554; *p* < 0.001), research experience (*β* = 0.287; *p* < 0.001), and the following scientific publications (*β* = 0.092; *p* = 0.009) were positively associated with EBP competence (*R* ^2^ = 0.643)	Nurses emphasized that education, critical thinking, and exposure to research activities strengthened their competence. ‘…as we gain experience, we start to research how to do this practice better…’ (G4, P18)	The regression results are illuminated by qualitative findings, which explain how education and professional engagement provide opportunities to develop deeper analytical skills and confidence in applying EBP
Clinical nurse position (*β* = −0.081; *p* = 0.020) and working in inpatient units (*β* = −0.083; *p* = 0.014) negatively predicted EBP competence (*R* ^2^ = 0.643)	Nurses described workload, insufficient staffing, and limited resources as barriers. ‘…we are so busy… it is difficult to access the most up‐to‐date evidence.’ (G3, P13)	Quantitative associations of lower EBP competence among clinical and inpatient nurses are clarified by qualitative evidence pointing to structural barriers such as heavy workload, insufficient staffing, and limited resources. This integration highlights how organizational factors reduce opportunities to practice EBP
Education (*β* = 0.275; *p* < 0.001), EBP competence (*β* = 0.603; *p* < 0.001), and research experience (*β* = 0.173; *p* < 0.001) positively predicted CDM levels, while clinical nurse position (*β* = −0.071; *p* = 0.046) was a negative predictor (*R* ^2^ = 0.567)	Nurses explained that EBPs and nurses' research experiences facilitated CDM, improved standardization, and enhanced care quality. ‘…EBPs improve the quality of patient care, reduce infection risks, and positively affect patients' well‐being.’ (G1, P5). Also, Participants reported barriers. At the organizational level, heavy workload, insufficient staffing, lack of institutional resources, were noted as major obstacles to effective CDM ‘We already have too many patients and too few staff; in such conditions, it is almost impossible to focus on evidence‐based decision‐making…’ (G2, P8)	The statistical link between education, EBP, and CDM is strengthened by qualitative accounts illustrating that EBP provides a scientific foundation for decisions. The negative association for clinical nurses is further explained by their descriptions of resource and workload constraints

## 5 Discussion

In this mixed‐methods study, the competencies of nurses in EBP and CDM and the factors influencing them were examined. Nurses were found to use EBPs in patient care, basic nursing practices and managerial processes. Both EBP and CDM levels were associated with socio‐demographic, professional and organizational factors. Both quantitative and qualitative findings showed that high competence scores were consistent with nurses' reports of applying protocols in daily practice. The positive effects of education, research experience, and following scientific publications identified in the quantitative results were supported by qualitative accounts emphasizing professional development and critical thinking. Lower competence among clinical and inpatient nurses in the quantitative data was also explained by qualitative findings highlighting workload, insufficient staffing and limited resources.

### 5.1 Discussion on Nurses' EBP Competencies and CDM Skills

In this study, the competency levels of nurses towards EBPs were generally high, with the mean total score corresponding to 75% of the maximum possible value. However, this competency remained mainly at the attitude level, while the knowledge and skills were markedly lower, indicating that knowledge and practical application were insufficient compared to attitudes. In the interviews, nurses also mentioned that they lacked knowledge about EBPs. Similar to this study, a systematic review conducted by Adombire et al. (2024) stated that nurses' attitudes towards EBPs were generally positive, but their lack of knowledge and skills prevented the integration of these practices into clinical practice. In addition, in this study, nurses had high levels of CDM, with the mean CDMNS total score corresponding to approximately 80% of the maximum value. Among subscales, nurses scored particularly high in evaluating results, but lower in searching for and adopting new information impartially, demonstrating strengths in outcome evaluation but weaknesses in analytical and evidence‐seeking skills. This aligns with the qualitative findings, where nurses described difficulties in accessing up‐to‐date resources and relying on routines due to workload. This finding indicates that while nurses have strong CDM experience, their analytical thinking and research skills need to be improved. In Türkiye, where the research was conducted, the Nursing Regulation published in 2010 explicitly states that nurses are expected to plan, implement, evaluate and supervise nursing care based on evidence (Republic of Türkiye Official Gazette 2010). While this regulatory framework provides a formal basis for EBP, the gap between attitude and knowledge observed in this study suggests that regulatory recognition alone may not be sufficient to ensure full integration into practice.

In addition to these findings, in this study, there was a positive relationship between nurses' CDM levels and EBP competencies. This finding coincides with previous studies emphasizing the importance of using evidence‐based knowledge in the CDM process. Koota et al. (2021) found that mastery of EBPs enables nurses to make more accurate and faster decisions in clinical situations and increases patient care quality and safety. In the interviews, nurses supported this finding and mentioned that EBPs are effective in making scientific decisions, which increase patient care quality and safety by standardizing nursing care. In addition, nurses also mentioned that EBPs support their professional image positively by supporting their professional development. These findings suggest that strategies should be developed to increase the knowledge level of nurses regarding EBPs, which affect patient care quality, patient safety and professional development. Regular training programs for nurses, hands‐on learning opportunities and institutional policies to facilitate access to research should be implemented (Jun et al. 2016; Koota et al. 2021; Sapri et al. 2022). In addition, it is critical to support nurses' critical thinking skills to use evidence more effectively in CDM processes (Melnyk and Fineout‐Overholt 2022; Schmidt and Brown 2024).

### 5.2 Discussion on Factors Influencing EBP Competencies and CDM Skills

According to the study's findings, nurses' EBP competencies and CDM levels are affected by individual and professional characteristics such as age, marital status, educational level, working unit, and professional experience. For instance, nurses aged 28 years and older and those who were married had higher scores on both EBP‐COQ and CDM. Nurses also mentioned that they made more evidence‐based decisions with professional experience during the interviews, and nurses with postgraduate education expressed that they felt more competent towards EBPs. The quantitative findings confirm this, as higher education was one of the strongest predictors of both EBP competence and CDM. In the literature, findings parallel with this study frequently emphasize that increasing the level of education of nurses improves their ability to access professional knowledge, evaluate knowledge and transform it into practice (Alatawi et al. 2020; Jun et al. 2016).

In this study, nurses' working units play a decisive role in the development of EBPs and CDM skills. Working in a clinical nurse position and in inpatient units reduced their competence levels. Similarly, working in inpatient units negatively affects nurses' EBP competencies due to limited resources and high patient density (Alatawi et al. 2020; Crawford et al. 2023). In addition, intensive care nurses have higher levels of competence and CDM. This finding can be interpreted as the necessity to make fast and accurate decisions, which increases the CDM competencies of nurses working in intensive care units, as they usually encounter more complex and critical patient cases. The literature states that intensive care nurses generally have more technical knowledge, exhibit advanced decision‐making processes, and, therefore, have higher awareness of EBPs (Šabanė et al. 2022; Yuliani 2023).

Nurses who spoke a foreign language, received training on EBP, regularly followed professional scientific publications, participated in scientific activities, and had experience in research planning or implementation scored significantly higher on EBP‐COQ and CDMNS. In the interviews, nurses mentioned that individual factors such as having critical thinking skills, knowing a foreign language, and having an education are effective in EBP competencies. These findings suggest that foreign language knowledge facilitates nurses' access to international literature and increases their EBP knowledge and skills (Yue et al. 2018). Training on EBPs and participating in scientific activities improves nurses' ability to evaluate and apply scientific knowledge (Koota et al. 2021; Sapri et al. 2022). Regularly following professional scientific publications supports nurses in making evidence‐based decisions and increases their self‐confidence (Ferreira et al. 2022). In addition, taking part in research planning and implementation processes supports nurses in making clinical decisions and strengthening their analytical thinking and critical approach skills (Melnyk and Fineout‐Overholt 2022).

In this study, nurses working in shift systems and those working 48 h or more per week had lower EBP and CDM competence. These findings were reinforced by qualitative accounts, where nurses described how adverse working conditions, insufficient staffing and high workload hindered their ability to use EBPs in care and CDM processes. A few nurses also reported individual barriers, such as low motivation and negative attitudes towards EBPs. Various studies have emphasized that long working hours and poor conditions increase burnout, reduce decision‐making quality, and undermine motivation to adopt EBPs (Alatawi et al. 2020; Crawford et al. 2023). Accordingly, organizational arrangements should be made to minimize workload, innovative strategies should be implemented to facilitate motivation and access to EBPs, and policies that improve working conditions should be developed to support nurses in adopting EBPs.

### 5.3 Strengths and Weakness

This study's strengths included using a mixed‐method design, combining quantitative and qualitative data collection, and identifying factors influencing nurses' decision‐making and EBP competencies through regression analysis. However, some limitations existed. The reliance on self‐reported questionnaires in the quantitative phase may introduce biases, such as social desirability or inaccuracies in self‐perception. To mitigate this, validated and widely used instruments (EBP‐COQ and CDMNS) were employed. The cross‐sectional design captures attitudes and competencies at a single time point, limiting insights into changes over time. While offering in‐depth perspectives through focus group discussions, group dynamics may have influenced the qualitative phase, with dominant participants overshadowing others. This risk was reduced by employing a trained moderator who encouraged balanced participation. The study's specific sample group and organizational context may also restrict the findings' generalizability. Nonetheless, qualitative data collection continued until data saturation was reached, and two independent coders analysed the transcripts to enhance credibility. Future research in diverse settings with broader samples could improve the applicability and relevance of these results.

## 6 Conclusion

This study revealed that individual, professional and organizational factors influence nurses' EBP competencies and CDM levels. While nurses utilize EBPs in patient care and managerial decisions, gaps in knowledge and implementation barriers persist. Nurses generally demonstrated high CDM levels, particularly in result evaluation, but skills in impartial adoption and research of new information need enhancement. Strategies should focus on integrating EBP competencies with CDM, promoting lifelong learning, addressing organizational barriers such as shift work and long hours and encouraging participation in scientific activities and research processes. Regular and comprehensive training programs that support EBP should be planned and implemented. These programs will support nurses in using scientific evidence more effectively in their professional practice by increasing their level of knowledge. Establishing technological infrastructure and support mechanisms that facilitate access to scientific publications and research results can improve nurses' access to up‐to‐date information and their ability to use it in practice. Future studies are needed to compare nurses' competencies and CDM levels working in different health institutions and units towards EBPs. In addition, it is recommended that comprehensive studies be included examining the effect of nurses' working environments and conditions on EBPs and experimental designs evaluating the impact of individual and organizational interventions in these processes.

## Author Contributions

**Seda Sarıköse:** data curation, formal analysis, investigation, methodology, project administration, writing‐original draft. **Fatma Sevim:** conceptualization, data curation, investigation, methodology, project administration, writing‐original draft. **Şebnem Alık:** data curation, investigation, methodology. **Pelin Karaçay:** conceptualization, investigation, project administration. **Özgen Yaşar:** conceptualization, investigation, project administration.

## Funding

This study was funded by the TÜBİTAK 2209‐A Research Project Support Programme for Undergraduate Students (Project number: 1919B012405188).

## Disclosure

The statistics were checked prior to submission by an expert statistician (Atilla Bozdoğan: atillabozdogan@gmail.com).

## Ethics Statement

The study was approved by the Koç University Committee on Human Research (Approval number: 2024.169.IRB3.076).

## Conflicts of Interest

The authors declare no conflicts of interest.

## Data Availability

The data that supports the findings of this study are available on request from the corresponding author. The data are not publicly available due to privacy or ethical restrictions.
